# Microsurgical Reconstruction with and without Microvascular Anastomosis of Oncological Defects of the Upper Limb

**DOI:** 10.3390/healthcare12202043

**Published:** 2024-10-15

**Authors:** Valentina Pinto, Flavia Zeneli, Pietro Giovanni di Summa, Gianluca Sapino, Davide Maria Donati, Fabio Bernagozzi, Riccardo Cipriani, Giorgio De Santis, Marco Pignatti

**Affiliations:** 1 Plastic Surgery, Azienda Ospedaliero-Universitaria di Modena, Via del Pozzo 71, 41125 Modena, Italy; valentina.pinto@unimore.it (V.P.);; 2 Department of Medical and Surgical Sciences (SMECHIMAI), University of Modena and Reggio Emilia, 41121 Modena, Italy; 3 Plastic Surgery, IRCCS Azienda Ospedaliero-Universitaria di Bologna, Via Albertoni 15, 40138 Bologna, Italy; 4 Department of Plastic and Hand Surgery, University Hospital of Lausanne (CHUV), University of Lausanne, Rue du Bugnon 46, 1011 Lausanne, Switzerlandgianluca.sapino@chuv.ch (G.S.); 5 3rd Orthopaedic and Traumatologic Clinic Prevalently Oncologic, IRCCS Istituto Ortopedico Rizzoli, Via Pupilli 1, 40136 Bologna, Italy; 6 Department of Biomedical and Neuromotor Sciences (DIBINEM), Alma Mater Studiorum University of Bologna, 40126 Bologna, Italy; 7 Department of Medical and Surgical Sciences (DIMEC), University of Bologna, 40126 Bologna, Italy

**Keywords:** upper limb reconstruction, perforator flap, free flap, microsurgery

## Abstract

Introduction: The choice of the most adequate surgical technique for upper limb defects remains challenging. The aim of this article is to discuss the main microsurgical (pedicled or free) reconstructive options for the post-oncological reconstruction of different anatomical areas of the upper extremity. Materials and methods: We reviewed different reconstructive methods reported in the literature needing microsurgical expertise and compared them to our clinical experience, in order to provide further guidance in the choice of different flaps for upper limb soft tissue reconstruction. Six clinical cases, one for each anatomical district, are presented as examples of possible solutions. Results: We report the options available in the literature for post-oncologic upper limb reconstruction, dividing them by anatomical area and type of flap: local flaps, regional flaps, free flaps, and distant pedicled flaps. Our examples of the reconstruction of each anatomical area of the upper limb include one reverse ulnar pedicled perforator flap, one free Antero-Lateral Thigh (ALT) flow-through flap, one perforator-based lateral arm flap, two myocutaneous latissimus dorsi pedicled flaps, and one parascapular perforator-plus flap. Conclusions: In oncological cases, it is important to consider reconstructive options that provide stable tissue and allow for the early healing of the donor and recipient site if the patient needs to undergo adjuvant radiotherapy or chemotherapy. A wider range of flap options is essential when choosing the proper technique according to the patient’s needs, surgeon’s preference, and logistical possibilities. Perforator flaps combine the advantages of other flaps, but they require microsurgical expertise. Free flap reconstruction remains the gold standard to obtain a better overall and cosmetic outcome in complex and wide defects, where no suitable local pedicled flap option exists. The pedicled latissimus dorsi flap should still be included among the reconstructive options for its strong vascularization, size, and arc of transposition.

## 1. Introduction

Soft tissue loss of the upper extremity may be caused by trauma, oncological resection, infection, burn injuries, or congenital malformations. The main goals of treatment are to provide early and stable wound closure, to restore function, and to preserve the esthetic appearance of the limb. Despite the multitude of reconstructive options described in the literature (skin grafts, pedicled local, regional and distant flaps, free flaps, and perforator flaps), the choice of the proper surgical technique remains challenging. The surgeon should consider the etiology, the size and location of the defect, the availability of local tissue and other local damage, the patient’s prognosis and comorbidities, the donor site morbidity, the clinical setting, and their personal experience.

Pedicled perforator flaps harvested using a microsurgical technique can represent a valid first reconstructive option, limiting the use of free flaps with micro-anastomosis to selected cases [1]. Specific traditional workhorse flaps, due to their reliability and the speed of their dissection, should not be excluded from the possible options, especially in the oncologic patient.

The aim of this article is to offer a standardized method of reconstruction for each anatomical area of the upper extremity, with a particular focus on oncologic patients.

## 2. Materials and Methods

Articles published between 2010 and 2023, describing upper limb reconstruction with different flaps, were selected from the Pubmed database. We reviewed the reconstructive methods reported in the literature [2,3,4,5,6,7,8,9,10,11,12,13] and compared them to our clinical experience to provide further guidance on upper limb soft tissue reconstruction. The upper limb anatomic units that were considered are as follows: hand, wrist, forearm, elbow, arm, and axilla.

**Figure 1:**
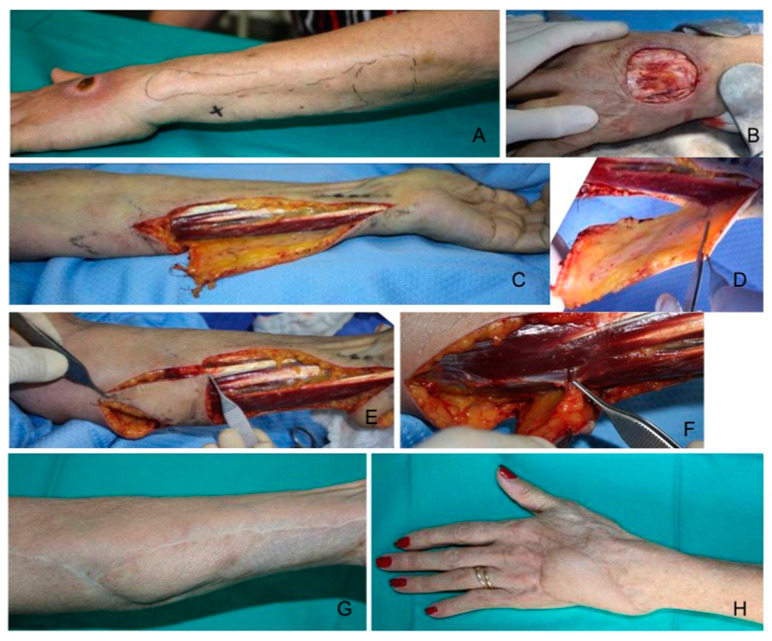
An 80-year-old lady affected by breast cancer sustained a soft tissue necrosis of the dorsum of the hand due to chemotherapy extravasation. (**A**) Skin integrity restoration was needed short-term to continue the treatments. After debridement (**B**), a reverse ulnar pedicled perforator flap (Becker flap) was harvested (**C**,**D**) to cover the medium-sized defect of the dorsum of the hand. The donor site defect (**E**) was partially restored with a propeller flap based on a proximal perforator of the ulnar artery (**F**). After 12 months’ follow-up, good healing was obtained on the hand (**H**) and donor site (**G**).

**Figure 2:**
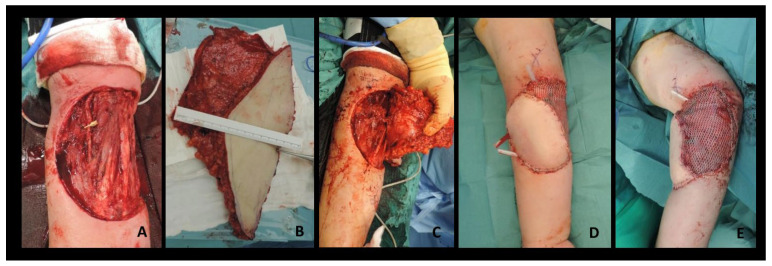
A 59-year-old man presented with a high-grade soft tissue sarcoma of the volar radial aspect of the proximal forearm. After tumor resection, the soft tissue defect was 20 cm × 18 cm, involving 80% of the circumference of the proximal forearm and including a 4 cm segment of the radial artery. (**A**) Considering the complexity of the defect, a free fasciocutaneous ALT flap was harvested, including the fascia lata. (**B**) The flap was used as a flow-through flap, and anastomosis was performed between the descending branch of the Lateral Circumflex Femoral Artery (LCFA) and the resected radial artery, both proximally and distally. (**C**) The fascia lata was covered by meshed split-skin grafts. (**D**,**E**) The donor site was closed primarily. The patient underwent adjuvant radiotherapy. During the 5-year follow-up, no complications occurred.

**Figure 3:**
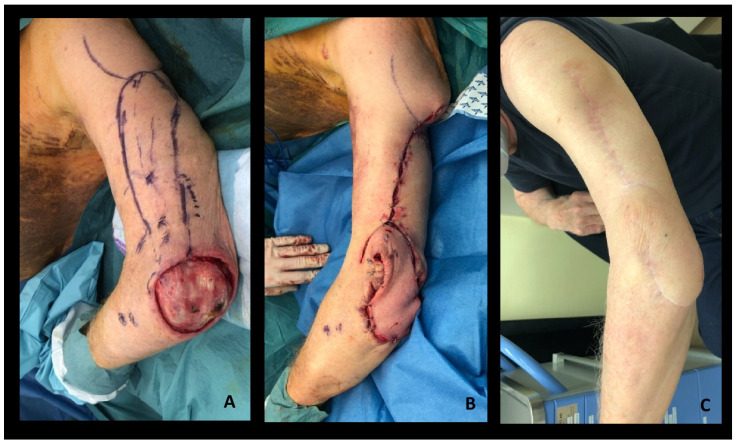
A 77-year-old man underwent excision of a stage III Merkel cell carcinoma involving the posterior aspect of the elbow. A wide local excision left a defect of 10 × 6 cm, with exposure of the olecranon bone. The perforators of the radial collateral artery perforator (RCAP) were found with the handheld Doppler. The skin paddle was designed, including the perforators. (**A**) The axial flap length was measured to be at least equal to the length from the pivot point to the edge of the defect. Dissection was performed in a subfascial plane and the course of the more distal reliable perforator was followed, up to its origin from the posterior radial collateral artery (PRCA). A skin bridge over the pedicle at the distal margin of the flap was maintained to improve the venous drainage (propeller plus), and the flap was rotated 180° into the defect. (**B**) The donor site was closed primarily. Postoperative radiotherapy and immunotherapy were performed. The patient had no complications and no movement limitations at a 25-month follow-up (**C**).

**Figure 4:**
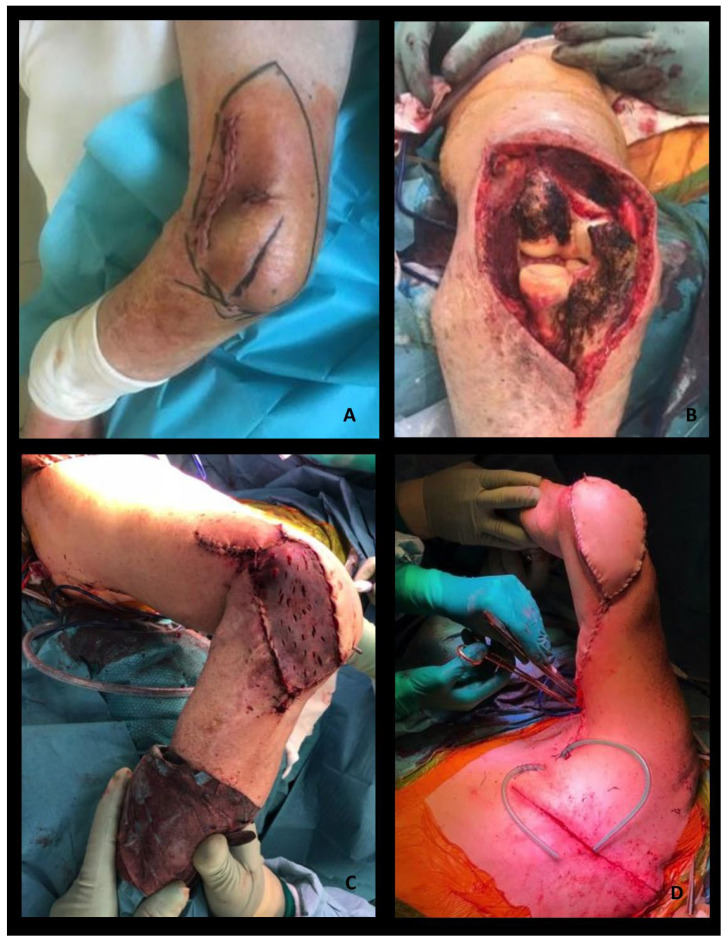
An 89-year-old man was referred to us for recurrence of sarcoma of the left elbow. Previous surgeries and adjuvant radiotherapy had caused extensive scar retraction, severe contraction, and stiffness of the elbow, with an 80% reduction in upper extremity function. (**A**) After a further surgical wide excision and the prosthetic joint replacement of the elbow (**B**), an ipsilateral Latissimus Dorsi (LD) myocutaneous pedicled flap was transposed for arthroplasty coverage and soft tissue reconstruction. The myocutaneous flap was harvested with a transverse skin island (19 cm × 10 cm). (**C**,**D**) The thoracodorsal pedicle was isolated to its origin to increase the flap transposition. The thoracodorsal nerve was preserved to ensure muscle trophism and adequate muscular thickness. A subcutaneous tunnel under the skin of the axilla and medial aspect of the left arm was dissected to transpose the flap on the wide surgical defect. LD muscle was used to cover the elbow prosthesis. The bulkiness of the skin paddle was used to reinforce the coverage of the prosthesis. (**D**) A split thickness skin graft was needed to completely cover the transposed LD muscle. (**C**) To avoid pedicle compression or strain, postoperatively, the shoulder was immobilized in 45 degrees of abduction for 3 weeks. Postoperative rehabilitation improved the final outcome and the joint function recovered well. Three months after surgery, the patient died of an acute ischemic stroke.

**Figure 5:**
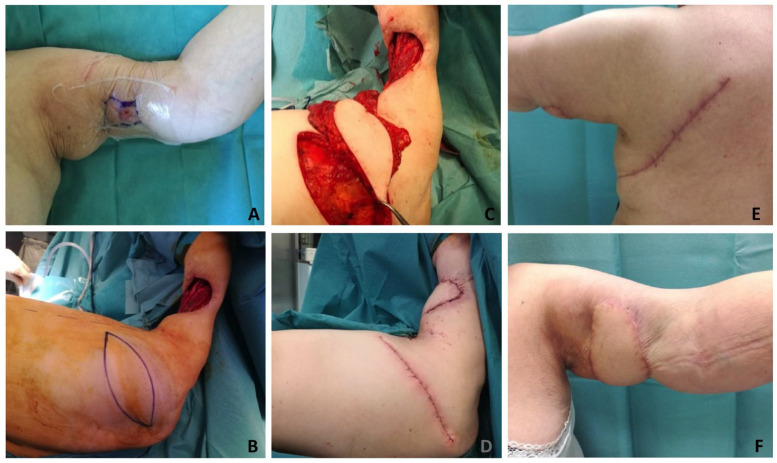
A 74-year-old woman, who had previously undergone surgical resection and adjuvant radiotherapy, presented with recurrence of leiomyosarcoma of the medial aspect of the left arm. (**A**) After a further surgical wide excision, an ipsilateral LD myocutaneous pedicled flap was transposed for radial nerve and brachial vessels coverage and for soft tissue reconstruction after biceps brachii and coracobrachialis muscle sacrifice. (**B**) The myocutaneous flap was harvested with a transverse skin island (21 cm × 9 cm). (**C**) The thoracodorsal nerve was preserved to maintain muscle trophism. The flap was tunneled under the axillary skin bridge to reach the arm defect (**C**), restoring adequate upper limb contour. (**D**) Postoperatively, the shoulder was immobilized in 45 degrees of abduction to avoid pedicle injury. Physical rehabilitation started 45 days after the reconstructive surgery, reporting more than 140 degrees of left arm abduction. The postoperative stay was uneventful, and the patient kept a stable soft tissue reconstruction at a 3-year follow-up, without recurrence (**E**,**F**).

**Figure 6:**
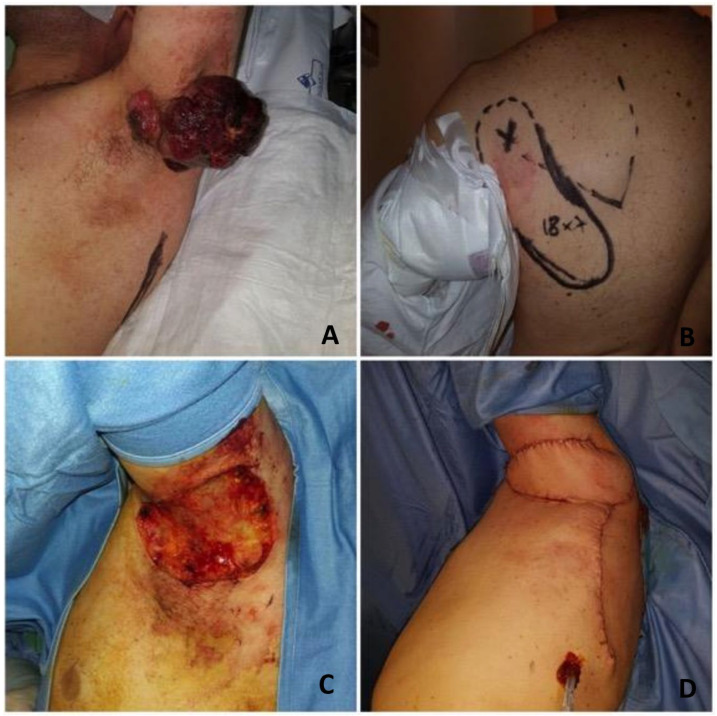
A 50-year-old man presented with a large exophytic ulcerated melanoma metastasis of the left axilla after the previous removal of cutaneous melanoma of the dorsum with positive sentinel node and axillary clearance refusal, 2 years before (**A**). The parascapular artery perforator was found with the handheld Doppler and the flap was marked preoperatively. (**B**) Palliative removal of the metastasis in continuity with the axillary lymphadenectomy was performed. (**C**) Strict adherence to the axillary vessels was present and the reconstruction of the medium-sized soft tissue defect of the axilla was achieved with a parascapular perforator-plus flap. (**D**) Medical treatment of the melanoma was started.

In addition, we selected and described six clinical cases, to further support evidence in the literature, among patients treated at the authors’ institutions for moderate to severe defects that required a more complex and interesting reconstruction. One case for each anatomical unit is shown (Figure 1, Figure 2, Figure 3, Figure 4, Figure 5 and Figure 6). Furthermore, we decided to focus on oncological patients because the need for further medical treatments results in a demand for successful reconstruction and early healing at first-stage surgery, making the decision process more challenging.

Preoperative antibiotic prophylaxis was administered in all cases. Low-molecular-weight heparin was administered until 7 days post-operatively in all cases as a prophylaxis for deep vein thrombosis and pulmonary embolism, but there is still no evidence on the benefits of anticoagulant or antiplatelet use in the prophylaxis of thrombosis of microvascular anastomosis. Informed consent was obtained from all patients.

## 3. Results

Six clinical cases which represent defects from different anatomical units (Figure 1, Figure 2, Figure 3, Figure 4, Figure 5 and Figure 6) are presented as an example of the possible solutions, as follows: an ulnar pedicled perforator flap (Becker flap) for the dorsum of the hand (Figure 1), a free ALT flap for the volar radial aspect of the proximal forearm (Figure 2), a propeller plus flap based on the perforators of the RCAP for the posterior aspect of the elbow (Figure 3), an LD myocutaneous pedicled flap for the elbow (Figure 4), an LD myocutaneous pedicled flap for the medial aspect of the arm (Figure 5), and a parascapular artery perforator flap for the axilla (Figure 6).

## 4. Discussion

In the literature, different surgical options and algorithms [2,3,4,5,6,7,8,9,10,11,12,13] can be found for the reconstruction of upper limb defects, without adequate differentiation of the etiology of the defects.

Table 1 shows a summary of the surgical options available in the literature for the reconstruction of different anatomical areas. Reconstructive options for adjacent anatomical units are similar; therefore, we grouped them into three major areas in Table 1.

### 4.1. Hand and Wrist

We will focus on dorsal hand reconstruction, as the reconstruction of the fingers and palm will not be discussed in this article. The dorsal skin is thin, pliable, and slightly redundant to allow wrist and finger movement. The subcutaneous tissue is minimally represented, and it glides over the tendons due to a smooth microvascular connective tissue.

Small-sized defects may be closed using direct suture, skin grafts, or local flaps (rotation, transposition, advancement). Small- to moderate-sized defects require either skin grafts or the mobilization of locoregional perforator flaps or axial flaps.

The first dorsal metacarpal artery (FDMA) flap is based on a perforator of the deep radial artery branch that arises between the tendon of the extensor pollicis longus and the first dorsal interosseous muscle. It supplies the radial dorsal skin up to the proximal phalanx of the II finger and is used for defects around the thumb and the first web space [14].

The dorsal metacarpal artery perforator (DMA, Quaba flap) flaps are elliptical-shaped flaps based distally at the second, third, and fourth intermetacarpal spaces. The arc of rotation up to 180° allows coverage of small defects over the metacarpals, web spaces, and the proximal phalanx [15]. They are thin, reliable, and allow primary closure of the donor site.

The radial artery perforator (RAP) flap can be raised both as a fasciocutaneous or adipofascial flap, based on the skin perforators arising from the radial artery at the level of the radial styloid. It easily reaches both dorsal and volar defects of the hand and wrist up to the metacarpal phalangeal joint (MCPJ), but the skin texture best matches the dorsal area. Its main advantage, compared to the radial forearm flap (RFF), is the preservation of the radial artery, thus reducing donor site morbidity. However, it has a smaller size and arc of coverage. Donor site closure often requires skin grafts [16].

The RFF can be harvested as a reverse flow pedicled flap to reconstruct the dorsal hand area. It is based on the retrograde flow through the ulnar artery and palmar arches after ligation of the proximal radial artery. This flap is reliable and does not require microsurgical dissection, but its main disadvantage over the RAP flap is the sacrifice of the radial artery [17,18].

The (dorsal) ulnar artery perforator (Becker flap, UAP) flap [19,20] is based on the distal perforator of the ulnar artery located 3 to 6 cm proximal to the pisiform bone. The potential flap size is 12 × 4 cm and, similarly to the RAP flap, it is used for the reconstruction of wrist, ulnar side, and dorsum of the hand, up to the MCPJ. Compared to the RAP flap, this flap provides relatively hairless skin and a more concealed incision; additionally, the donor site tends to heal better, as skin grafts are applied over muscle rather than tendons [2].

Distant pedicled and free flaps indications are limited to large or complex wound defects.

The main free flaps indicated for dorsal hand defects are RFF, lateral arm flap (LAF), medial sural artery perforator (MSAP) flap, dorsalis pedis flap (DPF), superficial circumflex iliac artery perforator (SCIP) flap [21,22], and anterolateral thigh (ALT) flap in thin patients or elevated with ultrathin dissection [23]. They provide thin pliable tissue and the pedicle length allows for microanastomosis away from the defect. The traditional ALT flap can lead to bulky results, depending on patient habitus, but can be subsequently thinned. Donor site morbidity remains low, although poor graft take may occur in DPF and RFF. The MSAP and SCIP flap dissection may be more demanding, and the vascular anatomy may vary. The general drawbacks of free flaps include a poor skin texture match, a longer operative time, and the need for microsurgical expertise [3].

Distant pedicled flaps described in the literature are mainly from the hypogastric and groin areas (superficial circumflex iliac artery—SCIA, superficial inferior epigastric artery—SIEA, deep inferior epigastric artery—DIEP, and paraumbilical perforators—PUP). Although they are reliable and easy to harvest, the need to immobilize the hand for up to 3 weeks limits their indications to specific cases, such as hand defects in children less than 2 years, soft tissue reconstruction prior to toe transfers, high-voltage electrical burns, and multiple hand and digital defects [24].

### 4.2. Forearm and Elbow

Small defects of the forearm can be closed directly or covered with skin grafts. Flap closure is reserved for large wounds or cases with exposed bone and tendons [4,6,7].

Elbow reconstruction requires a stable, yet pliable solution to enable early mobilization and prevent contracture and stiffness of the joint [4]. Therefore, flap coverage is the treatment of choice for small- to moderate-sized defects of the elbow.

The RFF can be used as a proximally based pedicled flap to cover defects of the proximal forearm and elbow. The radial artery is ligated distally, and the pivot point is at the cubital fossae.

The LAF is a septo-fasciocutaneous flap based on perforators of the posterior branch of the radial collateral artery (PRCA) and can be used as an antegrade (usually free) flap or retrograde (reverse) flap. In the latter case, the perfusion of the flap depends on the radial recurrent artery in the proximal forearm and is the most commonly used design for coverage of the posterior elbow region [25]. The proximal PRCA is ligated to enable the mobilization of the distally based pedicle and skin [26]. A skin bridge can be maintained at the pivot point to improve venous drainage and protect the pedicle from shearing (propeller plus flap) [27]. The other use of the LAF is as a free flap (antegrade flow), which, commonly in its extended version, includes a more distal skin island [28]. It is reliable, quick to harvest, and avoids the sacrifice of a major artery. The fascial component provides an optimal gliding surface for the underlying bone (or prosthesis) and neurovascular structures.

As described in the previous section, RAP and dorsal ulnar artery perforator flaps are often used in hand and wrist reconstruction. However, due to their wide range of motion as propeller flaps, they can also be used for small- to moderate-sized wounds of the distal forearm of the radial and ulnar regions, respectively.

The propeller flaps indicated for elbow reconstruction and adjacent areas, such as the proximal forearm and distal arm, are based on perforators of the following vessels, located just proximal to the elbow joint:

- Inferior ulnar collateral artery perforator (IUCAP);
- Superior ulnar collateral artery perforator (SUCAP);
- Brachial artery perforator (BAP);
- Radial recurrent artery perforator (RRAP);
- Radial collateral artery perforator (RCAP).

Flaps based on IUCAP, SUCAP, and BAP are located in the medial arm aspect and are indicated for anterior, medial, and posterior defects of the elbow. They provide hairless and more pliable skin than the lateral flaps and the donor site scar is less visible medially. RCAP- and RRAP-based flaps are located in the lateral arm aspect and are indicated for lateral elbow defects [5]. The perforator-based propeller flaps are thin, reliable, versatile, and provide an optimal texture match. Although microsurgical knowledge is required for dissection, they do not rely on microanastomosis and do not sacrifice major vessels [1].

Although not a perforator flap, the latissimus dorsi (LD) flap is commonly used for complex upper extremity reconstruction due to its large size, length of pedicle, range of rotation, strong vascularization, speed of dissection, and amplitude of indications. Based on thoracodorsal vessels, it can be used as a pedicled flap to cover large defects of the arm and elbow region or as a free flap in case of extensive forearm defects [7,8]. The predictable branching of the thoracodorsal artery allows splitting of the muscle to fill dead space and irregular defects. Furthermore, it can be used as a functional muscle transfer to restore elbow flexion and the external rotation of the shoulder [9]. Donor site morbidity has been reported to be tolerably low, due to the compensation of other shoulder muscles, although in paraplegic patient flaps, harvest can lead to disability. In addition, seroma formation on the donor site is common if quilting sutures are not applied during closure [6].

The thoraco-dorsal artery perforator flap (TDAP) is the perforator evolution of the LD flap. It has the advantage of minimizing donor site morbidity and preserving glenohumeral function, while maintaining the advantages of the long pedicle. The bulk of the flap is also reduced, at the price of a smaller surface and reduced coverage area.

Among free flaps for elbow reconstruction (several of which have already been discussed), the ALT flap provides pliable skin, a pedicle length up to 16 cm that enables the performance of a microsurgical anastomosis distant from the defect, and an acceptable donor site scar [8]. In non-thin patients, the ALT flap may be bulky for the elbow area. When needed, flap remodeling at a second stage can be offered, or thin or ultrathin dissection can be used to harvest the flap at the first operation [23].

### 4.3. Arm and Axilla

The greater laxity and availability of soft tissue in these areas allows most small- to moderate-sized defects to be successfully reconstructed by direct closure or local random flaps. Moderate- to large-sized defects or those with deep tissue loss require reconstruction with pedicled, perforator-based, or free flaps [10]. Most of the flaps used for forearm and elbow reconstruction can also be useful for defects of the arm.

The LAF is based proximally in these cases and can be rotated up to 180° to cover upper arm wounds [29]. If planned with an extended skin paddle (“extended” lateral arm flap—ELAF), the pedicle length enables the flap to reach the coracoid area or the axilla [30,31].

The propeller flaps based on IUCAP, SUCAP, BAP, RRAP, and RCAP vessels can easily cover the distal arm area, but their vascular territory may be included in the defect [5].

The parascapular flap is based on the descending branch of the circumflex scapular artery and can be harvested as a pedicled, propeller, or free flap. The pedicle length of up 7 cm allows it to reach the axilla without tension [32,33]. The advantages include constant vascular anatomy, a short operative time, a large flap size, and potential primary donor site closure with no functional deficit.

Furthermore, the LD and TDAP flaps are commonly employed to reconstruct this area, the details of which have been discussed previously. The serratus fascia and muscle can also be harvested on the same pedicle if required for bulk or more sophisticated reconstructions [9].

Other possibilities are represented by free flaps, which have already been mentioned previously, like ALT flaps or SCIP flaps. In the case of large skin defects, a DIEP flap has been used in the past with a super-thin harvesting to match the needs of the axillary reconstruction [34]. These flaps can provide a pliable and large amount of tissue but the need for microanastomosis increases the complexity of the surgical procedure; for this reason, their use is limited to large defects or if other pedicled flaps are unfeasible.

In our experience, the patient’s related factors, the clinical setting, and the surgeon’s expertise guide the choice of the best suitable surgical solution. Examples of the reconstruction of each anatomical area of the upper limb are reported in in our clinical cases (Figure 1, Figure 2, Figure 3, Figure 4, Figure 5 and Figure 6).

We used a reverse ulnar pedicled perforator flap for a medium-sized defect of the anterior aspect of the dorsum of the hand, a free ALT flow-through flap for a large defect of the forearm, a perforator-based lateral arm flap for a medium-sized defect of the elbow, a myocutaneous latissimus dorsi pedicled flap for a large defect over an elbow prosthesis, a myocutaneous latissimus dorsi pedicled flap for a large defect of the medial aspect of the left arm, and a parascapular perforator-plus flap for a medium-sized defect of the axilla.

A successful outcome of the reconstruction of the upper extremity defects requires stable and pliable coverage, the restoration of limb contouring, and functional recovery with the early motion of muscles, tendons, and joints.

The reconstructive strategy depends on the location and size of the post-oncological defect, previous radiotherapy or surgery, any needs for further therapy (radiotherapy or chemotherapy) and their timing, the possibility of planning staged procedures, and available resources.

Depending on the different etiologies of the defect, the choice of flap for reconstruction may change. Traumatic, burn, and electrical injuries often cause damage to the adjacent tissues and preclude the harvest of local flaps or microanastomosis to vessels close to the defect [9,10]. In oncological cases, it is important to consider reconstructive options that provide stable tissue and allow for early healing of the donor and recipient site if the patient needs to undergo adjuvant radiotherapy or chemotherapy [11]. In this context, although not true for perforator flaps, the pedicled radial forearm flap and latissimus dorsi flap have the advantage of reliability and fast harvesting and still have a role among the possible surgical options.

Sometimes, an intraoperative histological assessment can require a wider resection than previously planned. Having a wide range of flap options enables surgeons to overcome this problem.

The choice of flap can also depend on extrinsic factors such as patient compliance, hospital settings, and logistic limitations. The need to perform surgery in an external setting (i.e., a peripheral hospital), without adequate reconstructive planning (operative time, setting and instruments, and adequate postoperative monitoring) makes it necessary sometimes to rely on pedicled flaps rather than microsurgical flaps. An adequate compromise comprises pedicled perforator flaps, possibly with a propeller movement [35].

Perforator flaps combine the advantages of pedicled local flaps (with adequate color and texture tissue matches), pedicled regional flaps (a 180° arc of rotation), pedicled distant flaps (reliable), and free flaps (tissue located away from the zone of injury), and, in most cases, the donor site is closed primarily. Moreover, they offer quicker harvesting time and inset, when compared to a free flap. Although the flap dissection requires microsurgical skills, these flaps do not require a microvascular anastomosis (nor the related personnel expertise, instrumentation, and monitoring) [35].

However, the exact vascular territory perfused by a single perforator in vivo is unpredictable, and we prefer to use these flaps only for small- to medium-sized defects of the upper limb [5]. Whenever the defect cannot be reconstructed with a local flap, pedicled perforator flaps harvested using a microsurgical technique from the same limb or close-by areas are a valid reconstructive option.

According to the literature, free flap reconstruction is needed in 11–18% of patients undergoing limb-sparing surgery for upper extremity cancer, but needs expertise and adequate setting. Furthermore, previous radiotherapy increases the complication rate [36]. Free flap reconstruction remains the solution of choice in complex and wide defects, where no suitable local pedicled flap option exists.

The main limit of this study is that we did not conduct a systematic review of the literature according to PRISMA (Preferred Reporting Items for Systematic reviews and Meta-Analyses) guidelines. However, we believe that our work offers an extensive description of reconstructive options of the upper limb, combining information from the literature and the authors’ experience.

## 5. Conclusions

Many options for upper limb reconstruction have been described in the literature. Pedicled perforator flaps, with their multiple advantages, are our first choice for small- to medium-sized defects of the upper limb. For larger or more complex defects and in adequate clinical settings, free flaps should be considered. Selected traditional pedicled flaps should still be included among the reconstructive options, albeit mainly for small defects.

## Figures and Tables

**Table 1:** Reconstructive options for different anatomical areas. The main variable in deciding the reconstructive options is the size of the defect and its location in the different limb anatomical units. The surgical options are either classified as local flaps, regional flaps, free flaps, or distant pedicled flaps.

Type of Flap	Hand and Wrist	Forearm and Elbow	Arm and Axilla
Local Flaps	- FDMA - DMA	- RFF - LAF - RAP - UAP	- LAF - IUCAP - SUCAP - BAP - RRAP - RCAP
Regional Flaps	- RAP - UAP - Reverse RFF	- IUCAP - SUCAP - BAP - RRAP - RCAP - LD	- Parascapular Artery Flap - LD - TDAP
Free Flaps	- SCIA - SIEA - DIEP - PUP	- LD - ALT	- LD - TDAP - ALT - SCIP - Ultra-thin DIEP
Distant Pedicled Flaps	- RFF - LAF - MSAP - DPF - ALT - SCIP	-	-

Abbreviations: FDMA, first dorsal metacarpal artery; DMA, dorsal metacarpal artery; RAP, radial artery perforator; UAP, ulnar artery perforator; RFF, reverse radial forearm flap; SCIA, superficial circumflex iliac artery; SIEA, superficial iliac external artery; DIEP, deep inferior external artery; PUP, paraumbilical perforators; LAF, lateral arm flap; MSAP, medial sural artery perforator; DPF, dorsal pedis flap; ALT, anterolateral thigh; SCIP, superficial circumflex iliac artery perforator; IUCAP, inferior ulnar collateral artery perforator; SUCAP, superior ulnar collateral artery perforator; BAP, brachial artery perforator; RRAP, radial recurrent artery perforator; RCAP, radial collateral artery perforator; LD, latissimus dorsi; TDAP, thoracodorsal artery perforator.

## Data Availability

Data is contained within the article.
